# In Vivo Comparison of Retention, Maximum Bite Force, and Patient Satisfaction Between Different Attachment Systems Used in Implant-Retained Overdentures

**DOI:** 10.7759/cureus.114523

**Published:** 2026-08-14

**Authors:** Keerti Goyal, Pardeep K Bansal, Yashika Aggarwal, Akansha Gupta, Gursimran Purba, Preetika Bansal

**Affiliations:** 1 Department of Prosthodontics, Dasmesh Institute of Research and Dental Sciences, Baba Farid University of Health Sciences, Faridkot, IND; 2 Department of Periodontics, Dasmesh Institute of Research and Dental Sciences, Baba Farid University of Health Sciences, Faridkot, IND

**Keywords:** ball abutment, biting force, dental implant, mandibular overdentures, positioner abutment, retentive force

## Abstract

Background: There is limited clinical literature available that directly compares retention, maximum bite force, and patient satisfaction between ball and locator attachment systems. Therefore, it is of interest to compare retention, maximum bite force, and patient satisfaction between ball and self‑aligning (positioner) attachments in mandibular implant‑retained overdentures.

Materials and methods: Twenty edentulous patients were selected for the study and randomly divided into ball and self-aligning (positioner) attachment overdentures.

Results: Over follow‑up, the self‑aligning attachment group showed greater retention, higher bite force, and better patient satisfaction compared to the ball‑attachment group. Thus, we show that the self-aligning positioner attachments are relatively better than ball attachments in terms of retention, bite force, and patient satisfaction.

Conclusion: The present study provides valuable insights into the comparison of attachment systems for implant-supported overdenture; further research is imperative to validate these findings comprehensively. Additional clinical studies focusing on other attachment systems are crucial for advancing the field of implant dentistry and to provide a better understanding of long-term outcomes of these attachments.

## Introduction

Edentulism negatively affects quality of life by impairing oral function and causing residual ridge resorption. This resorption reduces alveolar bone height and denture-bearing area and is approximately four times greater in the mandible than in the maxilla. Conventional dentures are commonly used to restore lost teeth but may have limitations in retention and masticatory efficiency [1,2]. To overcome these problems, advancements in prosthetic dentistry have introduced implant-retained overdentures, which are removable dental prostheses supported by oral tissues and retained by dental implants designed to improve retention and masticatory function. An overdenture is defined as a removable prosthesis that covers and rests on one or more remaining natural teeth or dental implants [1]. Two-implant-retained mandibular overdentures should be considered the first-choice treatment for edentulous mandibles, as supported by the McGill and York consensus statements [1,2]. Evidence from randomized controlled trials and systematic reviews demonstrates significantly greater patient satisfaction, denture stability, masticatory function, and oral-health-related quality of life compared with conventional complete dentures. The two-implant approach also provides a predictable, cost-effective treatment while minimizing surgical and prosthetic complexity.

Implant-supported mandibular overdentures eliminate mandibular denture movement, and subjects with these overdentures have bite force magnitudes 60%-200% higher than those with traditional complete dentures. Clinically stable mandibular dentures are the most significant factor influencing patient satisfaction. Implant-retained overdentures increase patient satisfaction and minimize pain during chewing. Patient satisfaction varies with different attachment systems, and its impact on quality of life is assessed by the Oral Health Impact Profile. The attachment mechanism in implant overdentures provides enhanced retention and stability compared to conventional dentures [2]. Attachments are mechanical devices for the fixation, retention, and stabilization of prostheses. The type of attachment used influences the load delivered to the implant and the retention of the overdenture. Commonly used attachment systems include stud attachments (e.g., locator and ball attachments), bar-and-clip attachments, and magnetic attachments. For example, LOCATOR® attachments provide effective retention while allowing some degree of rotational movement of the overdenture, making them particularly suitable for two-implant mandibular overdentures. Ball attachments provide simple and reliable retention through a matrix that engages the spherical abutment attached to the implant. Bar attachments splint multiple implants and provide greater stabilization and support, particularly in cases where additional retention is required [2,3].

Magnetic attachments offer relatively simple insertion and removal and may be beneficial for patients with limited manual dexterity. The choice of attachment influences the amount and direction of load transmitted to the implants and surrounding tissues, as well as the retention, stability, maintenance requirements, and long-term performance of the overdenture. Retention depends on the attachment type, the direction of applied dislodging forces, inter-implant distance and implant quantity, placement, and alignment. Various attachment mechanisms are available. Ball attachments are considered the simplest type, being cost-effective, less technique-sensitive, less dependent on implant position, easier to clean and replace, and easier to adjust [3,4]. The newly developed locator system, introduced in 2001, gained popularity due to its self-locating features, ease of seating in the oral cavity, and ability to accommodate non-parallel implants up to 40 degrees of divergence. Subsequently, the LOCATOR R-Tx system, introduced in 2016, incorporated an improved retentive mechanism and increased pivoting capability, allowing accommodation of up to 60° of divergence between implants, thereby improving alignment and overdenture seating in cases with greater implant angulation. More recently, the LOCATOR Angled Abutment, introduced recently, was developed to address off-axis implant placement by providing additional angulation correction of up to 35°, combining abutment angulation with extended-range or fixed inserts. These developments have expanded the clinical applicability of locator-type attachments in patients with divergent or unfavorably angulated implants. LOCATOR attachments come in different colors (blue, orange, white) and have reduced height, advantageous for limited interocclusal space. This allows the clinician to select the appropriate retention according to the patient’s clinical requirements, implant angulation, and ease of denture removal. Blue inserts generally provide higher retention, whereas orange and white inserts provide progressively lower retention, allowing customization of overdenture retention [4,5]. Their low-profile design also makes LOCATOR attachments particularly advantageous in cases with limited interocclusal space. The locator device uses a dual retention approach and offers different retention values. It is available with inserts offering different levels of retention, allowing the clinician to select the appropriate retention according to the patient’s needs. This provides flexibility in treatment and facilitates easier insertion and removal of the prosthesis.

Few studies report that LOCATOR attachments possess higher retentive force compared to ball systems, while others report that ball attachments are more retentive based on patient perception [3]. Therefore, it is of interest to evaluate and report the retention, bite force, and patient satisfaction between ball and self-aligning LOCATOR attachment systems.

## Materials and methods

This in vivo study involved human participants and was conducted in the Department of Prosthodontics, Dasmesh Institute of Research and Dental Sciences, Faridkot. The study aimed to compare the retention, maximum bite force, and patient satisfaction between self-aligning and ball attachment systems used in implant-supported overdentures.

Ethical approval

Institutional ethical clearance (approval number: IRB/22, dated 23 May 2022) was obtained before commencement of the study.

Study design and duration

The study was designed as a prospective study. The study duration was from January 2022 to December 2026.

Sample size calculation

The sample size for the current study was calculated using G*Power 3.1.9.7 software (Heinrich-Heine-Universität Düsseldorf, Düsseldorf, Germany). For a t-test comparing the means of two independent groups (one-tailed), a confidence level of 34, a Z score of 0.4399, a margin of error of 4%, a margin of 0.04, and a proportion estimate of 20% resulted in a required sample size of 20 subjects [4]. To enhance the reliability and ensure adequate representation, the final sample size for the present study was rounded up to 20 subjects per group, thereby exceeding the minimum requirement suggested by the power analysis.

Inclusion criteria

Subjects aged ranging from 45 to 70 years with completely edentulous maxillary and mandibular ridges and good oral hygiene, absence of lingual exostosis and local inflammation, healed bone sites at least four months post-extraction, adequate residual bone height of a minimum of 10-15 mm in the anterior mandible region, and well-informed and motivated patients were included in the present study.

Exclusion criteria

Patients with any systemic or debilitating diseases, uncontrolled diabetes, blood dyscrasias, or immunological diseases such as AIDS, cirrhosis of the liver, irradiated cancer patients, and any patients having a habit of smoking were excluded from the present study.

Patient allocation and grouping

Twenty edentulous patients were chosen for the study and randomly divided into two equal groups. Randomization was performed using a computer-generated random allocation sequence. Each participant was assigned a unique identification number, and the sequence was used to allocate participants equally to either Group 1 or Group 2. Allocation was performed after completion of the baseline assessment.

Group 1

This group included patients in whom two conventional two-piece implants (SuperLine; Dentium Co., Ltd., Seoul, Republic of Korea) were placed in the interforaminal region of the anterior mandible, followed by fabrication of a mandibular implant-supported overdenture with ball attachments.

Group 2

This group included patients in whom two conventional two-piece implants (SuperLine) were placed in the interforaminal region of the anterior mandible, followed by fabrication of an implant-supported mandibular overdenture with a self-aligning (positioner) attachment system.

Patient’s consent

The patients signed a written consent form approving their treatment plan after being educated about the course of treatment and the necessity of frequent and regular recall.

Preoperative examination and investigations

A detailed general, physical, and clinical examination was performed for all 20 patients. Blood investigations, including hemoglobin, bleeding time, clotting time, viral markers, and random blood sugar (RBS), were conducted. An orthopantomogram (OPG) was also obtained for each patient.

Step 1: implant placement

A comprehensive pre-surgical protocol was followed for implant placement in the edentulous mandibular symphysis region. Each patient underwent thorough medical and dental evaluation, including intraoral assessment and diagnostic impressions for study models. Implant size selection was guided by ridge mapping using K-files and sectioned casts to assess bone availability, accounting for soft tissue thickness. Pre-medication included amoxicillin 500 mg administered prior to surgery and continued for five days. Patients were prepared with antiseptic rinses and draping, followed by local anesthesia. Surgically, a crestal incision was made, and a full-thickness flap rose and osteotomy performed under irrigation. Two implants were placed in the mandibular canine regions, ensuring appropriate torque and parallelism. The site was irrigated, sutured, and a pressure pack was applied. Postoperatively, patients were monitored with OPG imaging and prescribed antibiotics, analgesics, and chlorhexidine rinses. Sutures were removed after one week. After three months, stage II surgery was conducted to place healing caps, and patients were recalled for the prosthetic phase.

Step 2: prosthetic phase

After seven days of stage II surgery, prosthetic treatment was started. Healing caps were removed.

Diagnostic impressions were made using irreversible hydrocolloid (Zelgan; Dentsply India Pvt. Ltd., Gurgaon, India). Special trays were fabricated using autopolymerizing acrylic resin. Incremental border molding was performed using green stick compound, followed by wash impressions using addition polyvinyl siloxane light-body material (Zhermack S.p.A., Badia Polesine, Rovigo, Italy). Record bases and occlusal rims were fabricated. Jaw relations were recorded, the teeth were arranged, and a trial was performed intraorally. The dentures were processed using heat-cured acrylic resin, followed by finishing and polishing, and then inserted. Occlusal adjustments were subsequently performed.

Prosthetic loading

Group 1

**Figure 1:**
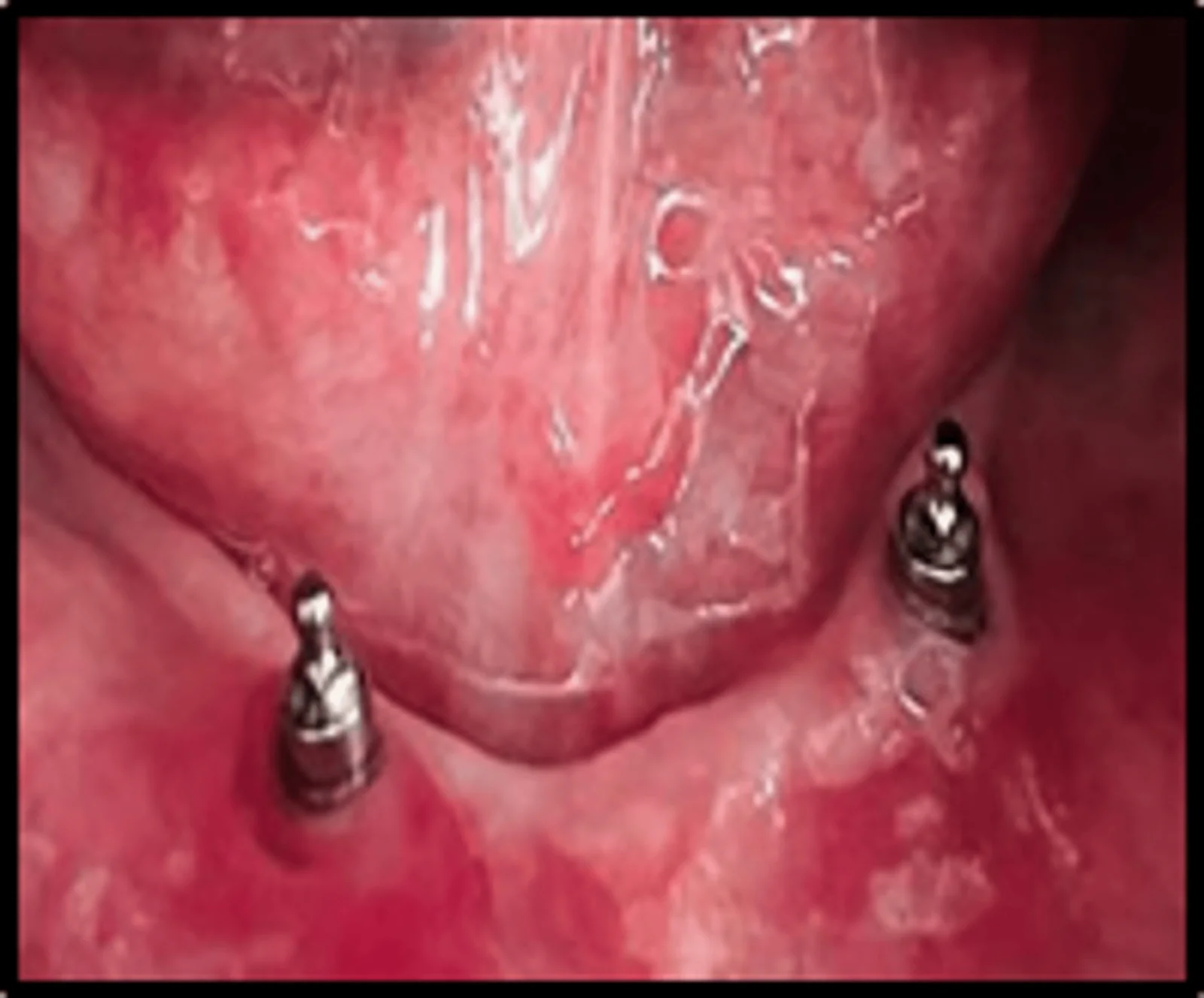
Ball abutments placed

Ball abutments (Figure 1) were tightened using hand torque and a torque wrench (20 N·cm). Female sockets were placed and picked up with autopolymerizing resin in the denture. To allow escape of excess resin, lingual vent holes were made. The denture was seated fully, excess resin trimmed, polished, and inserted. Patients were trained for insertion and removal, and appropriate home-care instructions were provided.

Group 2

**Figure 2:**
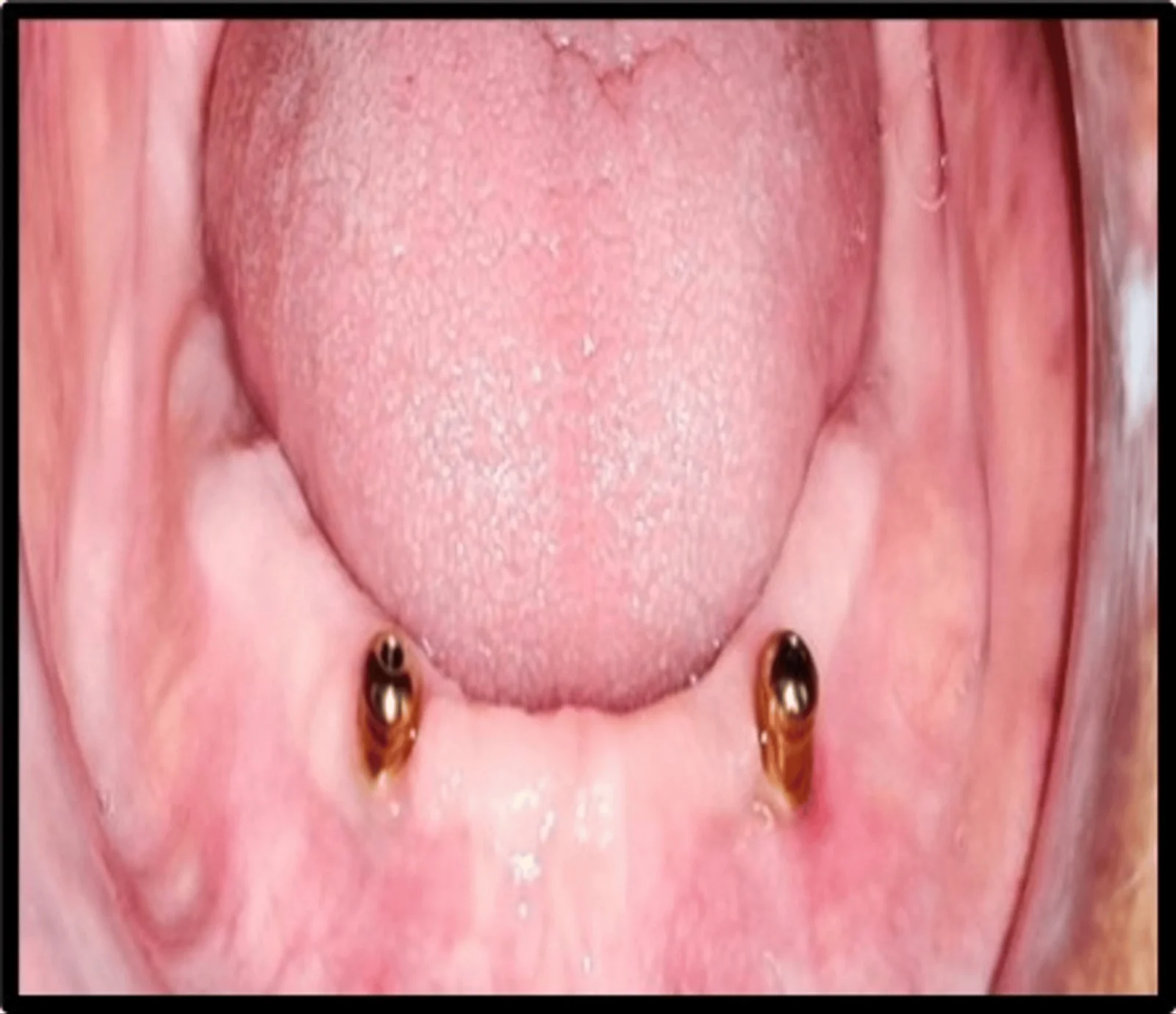
Positioner abutments placed

**Figure 3:**
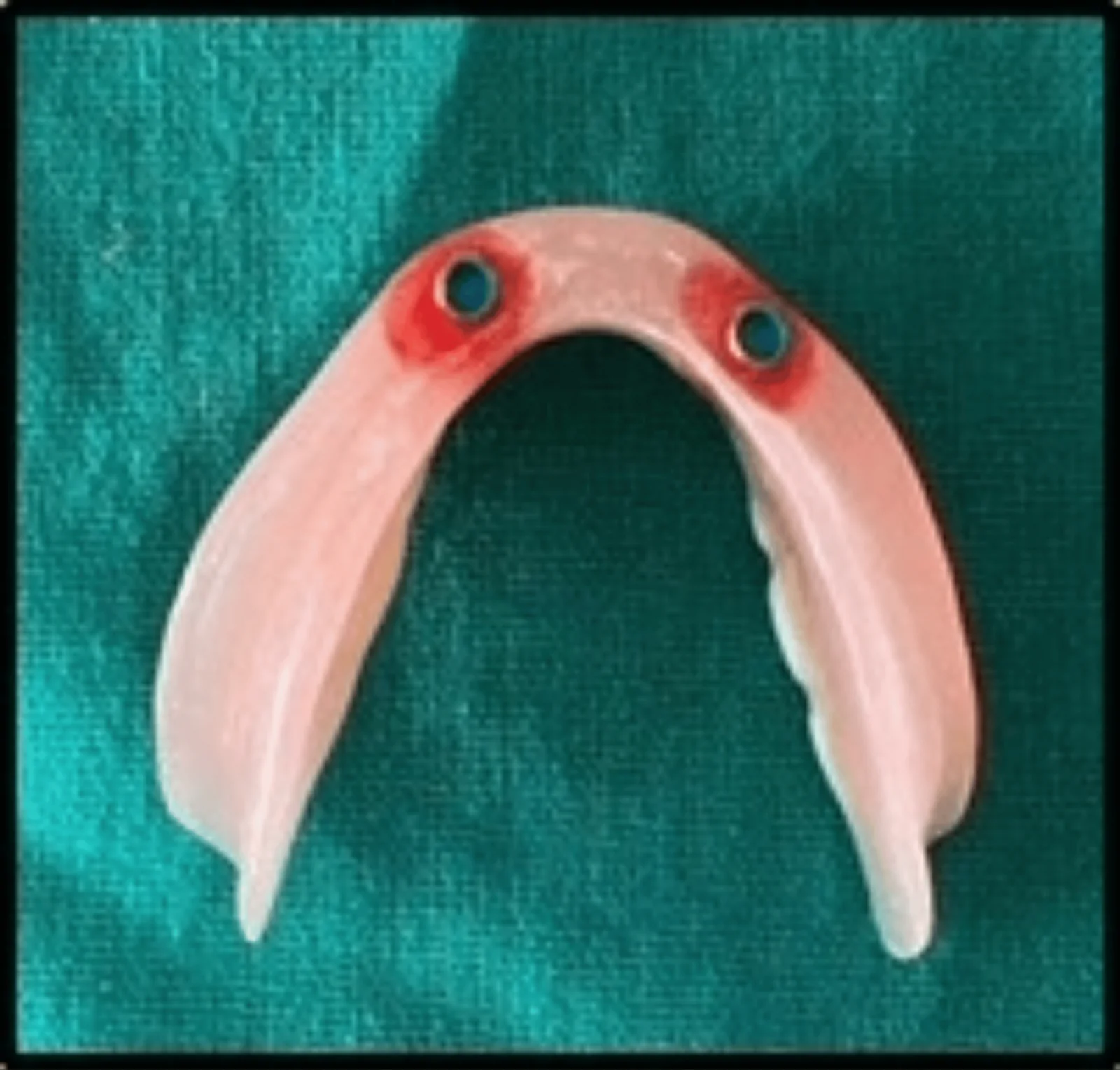
Pickup of attachment in the overdenture

Positioner abutments (Figure 2) were torqued at 20 N·cm, block-out spacers were placed, metal sockets were positioned, and the denture was relieved to accommodate the attachments. Lingual vents were created. Autopolymerizing resin (Figure 3) was used for pickup. After setting, the white processing male components were replaced with blue nylon inserts for retention. The denture was inserted, and the patient was trained to insert and remove the denture independently.

Analysis of retention and bite force before prosthetic loading

**Figure 4:**
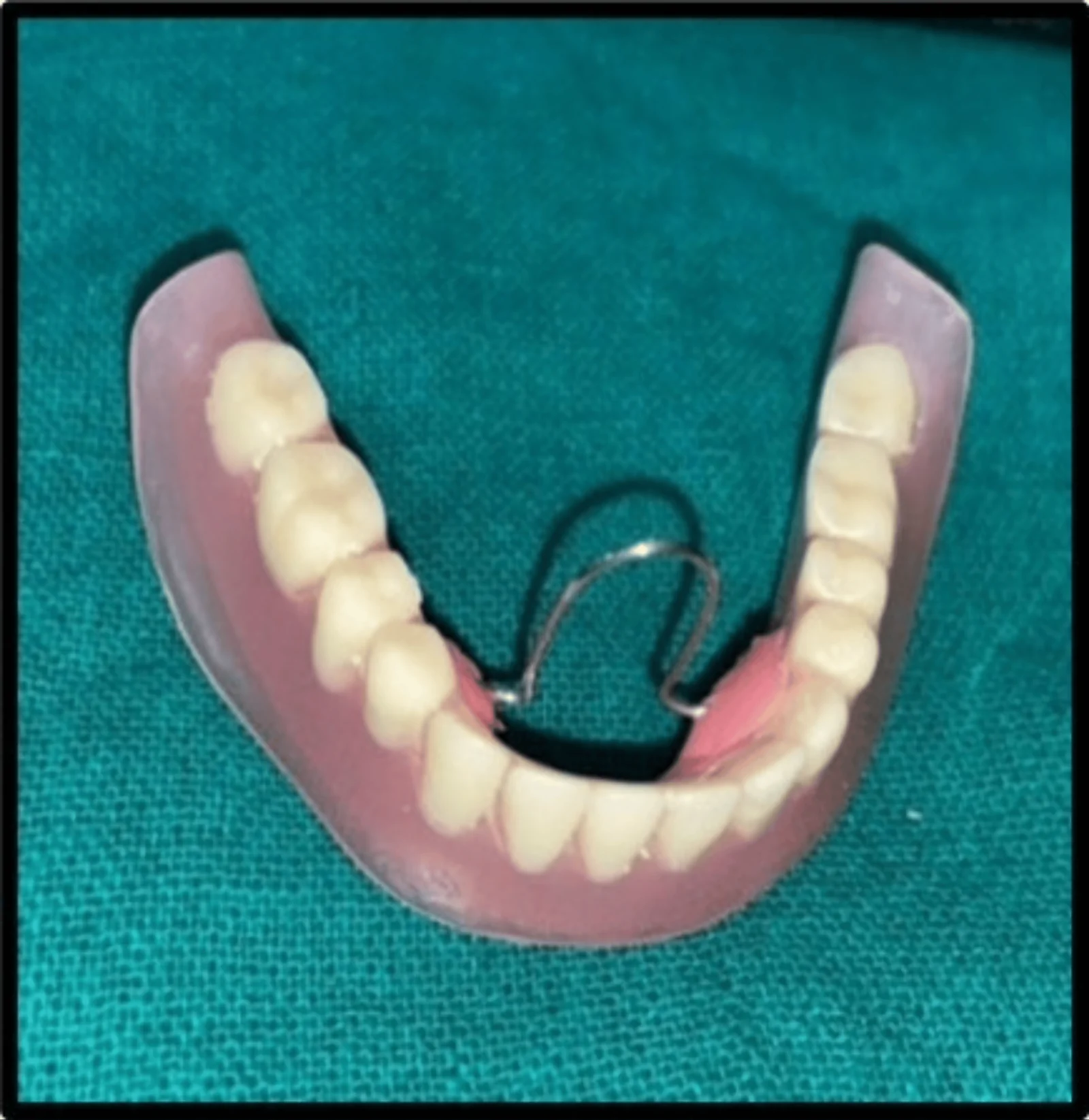
Wire hook attached to the denture for measuring retention

**Figure 5:**
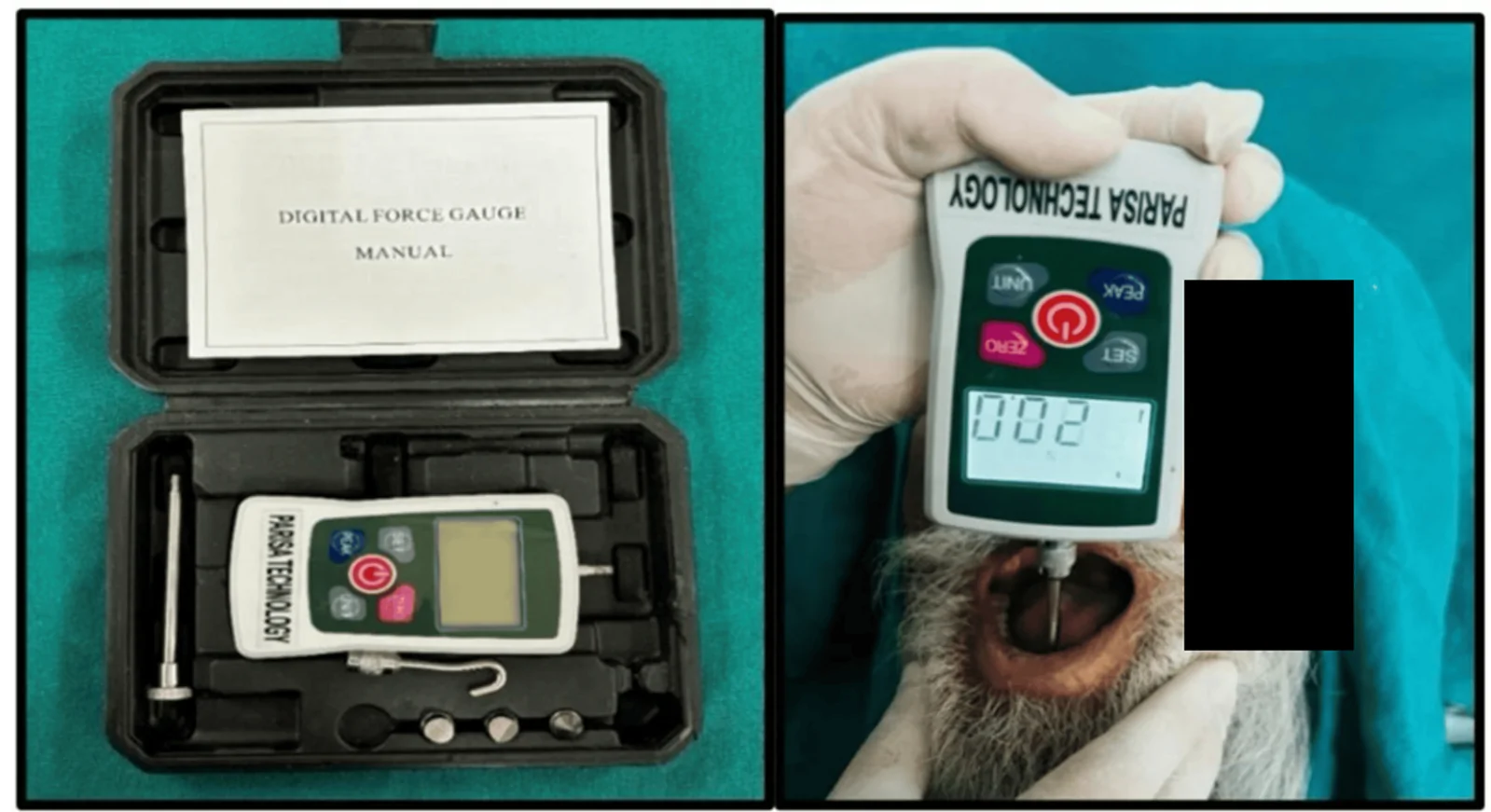
Retention measured using a digital force gauge

**Figure 6:**
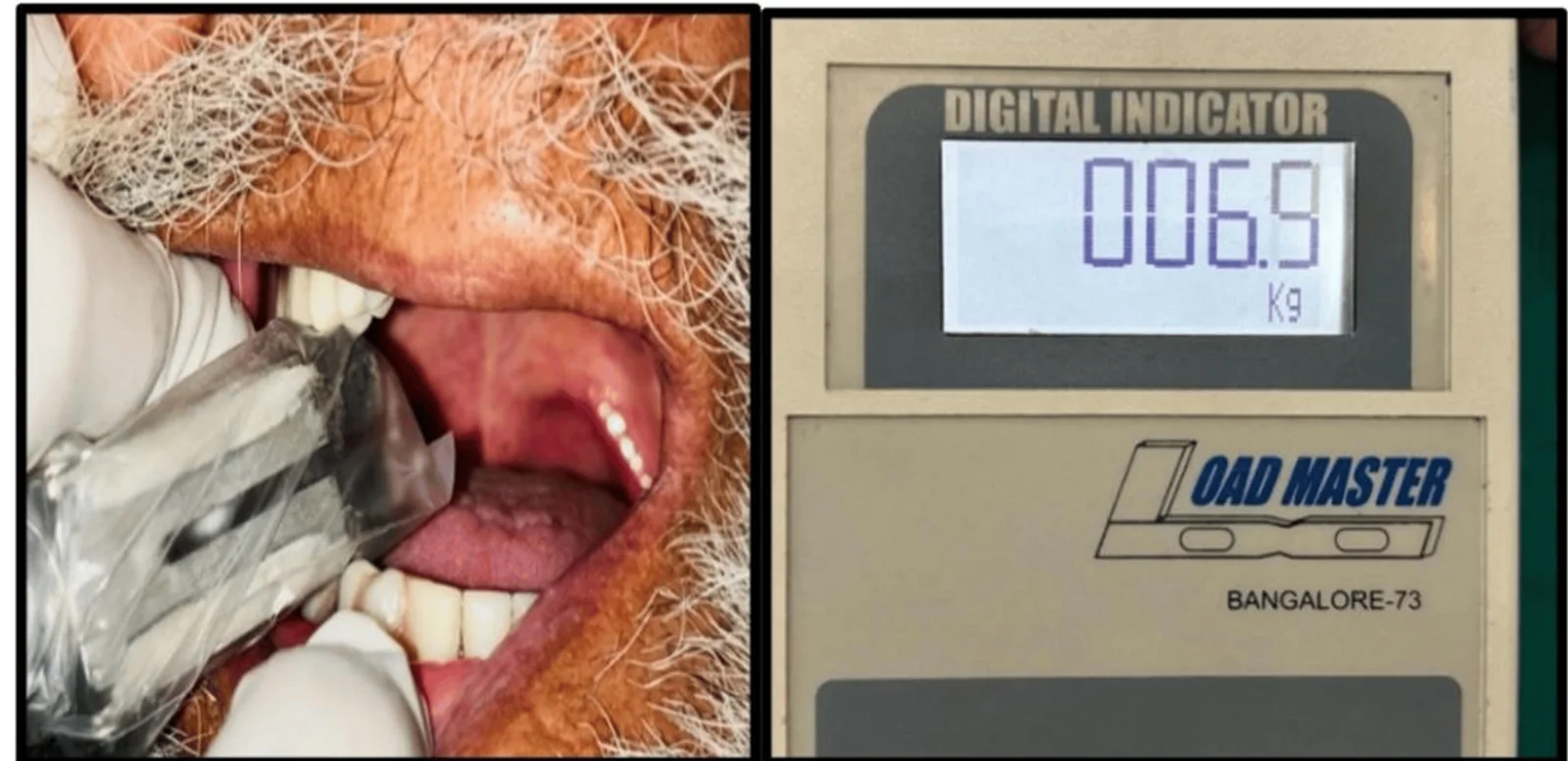
Bite force measured using a gnathodynamometer

Retention was measured using a digital force gauge. A 0.9-mm orthodontic wire hook (Figure 4) was attached to the lingual aspect of the anterior teeth using cold-curing acrylic resin. The shaft of the digital force gauge (Figure 5) was engaged with the hook, and a vertical pulling force was applied until the denture was dislodged. The force was recorded in Newtons, and the measurement was repeated three times. Bite force was recorded using a Gnathodynamometer (Load Master, Bangalore, India) (Figure 6).

Patients were instructed to bite on the device with the strain gauge covered with a disposable plastic cover to avoid cross-infection. Biting force was recorded bilaterally and charted. Patients were recalled at one week (T1), one month (T2), and three months (T3) after overdenture insertion. Retention and bite force were evaluated using the same procedures described above.

Retention is one of the primary objectives in impression making. It can be measured in conventional dentures as well as in overdentures.

Analysis of patient satisfaction

A 100-mm Visual Analogue Scale questionnaire, based on the method described by Awad et al. (2003), was used to evaluate patient satisfaction [6]. The scale ranged from complete dissatisfaction (0) to complete satisfaction (100). The questionnaire was administered to patients in both groups before prosthetic loading and at one week, one month, and three months after overdenture insertion. The data obtained were compiled, analyzed, and statistically compared.

Statistical analysis

Results obtained were subjected to statistical analysis using IBM SPSS Statistics for Windows, Version 26 (Released 2018; IBM Corp., Armonk, New York, United States). Data were analyzed for normal distribution using the Kolmogorov-Smirnov test and were found to be normally distributed. Therefore, a parametric test of significance was applied. Descriptive statistics were performed. Data were presented as mean and standard deviation. Intergroup comparison was done using an independent ‘t’-test. Intragroup comparison was done using a paired ‘t’-test. A p-value less than 0.05 was considered statistically significant. The effect size was chosen based on Cohen's guidelines, anticipating a substantial difference in the masticatory bite force between ball and flat attachments, ensuring the study is adequately powered to detect clinically meaningful differences.

## Results

The study evaluated retention force, bite force (left and right), and patient satisfaction across different time intervals in Group 1 and Group 2. Retention force increased significantly from baseline to one week in both groups, with Group 2 showing a higher increase. However, from one week to three months, changes were not significant within groups (Table 1). Retention and bite force are measured in terms of Newtons.

**Table 1:** Comparison of changes in retention force from baseline to three months between Group 1 and Group 2 An independent-samples t-test was used for statistical analysis. p < 0.05 was considered statistically significant.

Groups	Before loading (T0)	After one week (T1)	After one month (T2)	After three months (T3)	p-value (T0-T1)	p-value (T1-T2)	p-value (T2-T3)
Group 1	30.81 ± 0.653 N	54.99 ± 0.975 N	55.22 ± 0.814 N	55.36 ± 0.857 N	0.001 (sig)	0.946 (non-sig)	0.992 (non-sig)
Group 2	31.55 ± 0.552 N	63.58 ± 1.771 N	64.12 ± 1.850 N	64.28 ± 1.898 N	0.001 (sig)	0.941 (non-sig)	0.987 (non-sig)

Bite force on both sides showed a similar pattern, with significant improvement from baseline to one week and no significant change afterward. Group 2 consistently demonstrated higher bite force values than Group 1 through all follow-up periods (Table 2).

**Table 2:** Change in bite force (left side) from baseline to three months between Group 1 and Group 2 An independent-samples t-test was used for statistical analysis. p < 0.05 was considered statistically significant.

Groups	Before loading (T0)	After one week (T1)	After one month (T2)	After three months (T3)	p-value (T0-T1)	p-value (T1-T2)	p-value (T2-T3)
Group 1	1.90 ± 0.442 N	10.69 ± 0.64 N	10.36 ± 0.665 N	9.91 ± 0.657 N	0.001 (sig)	0.879 (non-sig)	0.923 (non-sig)
Group 2	2.04 ± 0.579 N	20.38 ± 1.076 N	19.77 ± 1.24 N	18.04 ± 1.04 N	0.001 (sig)	0.921 (non-sig)	0.938 (non-sig)

Patient satisfaction scores also increased over time in both groups, with Group 2 reporting significantly higher satisfaction at each interval. Overall, Group 2 showed superior performance in all measured parameters (Table 3).

**Table 3:** Comparison of patient satisfaction at different time intervals between Group 1 and Group 2 An independent-samples t-test was used for statistical analysis. p < 0.05 was considered statistically significant.

Groups	After one week (T1)	After one month (T2)	After three months (T3)	p-value (T1-T2)	p-value (T2-T3)
Group 1	53.95 ± 2.998 N	59.39 ± 2.251 N	65.53 ± 2.293 N	0.001 (sig)	0.001 (sig)
Group 2	66.23 ± 2.634 N	73.10 ± 3.044 N	79.26 ± 4.213 N	0.001 (sig)	0.001 (sig)

## Discussion

Edentulous patients have long been rehabilitated using traditional complete dentures; however, they are often associated with compromised retention and stability, especially in the mandibular arch. This leads to functional limitations such as difficulty in speech, mastication, and esthetics, and significantly impacts patient satisfaction. To address these issues, various methods like vestibuloplasty, bone grafting, ridge augmentation, specialized impression techniques, and adhesives have been employed, though with limited and inconsistent benefits [3-7]. In the early 1980s, when dental implants were introduced, many of these challenges were mitigated. As emphasized by the McGill and York consensus (2002), restoration of the edentulous mandible using conventional complete dentures is no longer considered the first-line prosthodontic treatment. Implant-retained overdentures have emerged as the preferred option, offering improved denture stability, retention, and support, thereby enhancing mastication, appearance, and quality of life [8,9]. In the present in vivo study, we compared the effectiveness of ball attachments (Group I) and self-aligning positioner attachments (Group II) in terms of retention, bite force, and patient satisfaction. The retention forces were significantly higher in Group II at all intervals, which supports the results of Upinder et al. (2019), who demonstrated prolonged function and greater retention with locator-type attachments in in vitro studies [10]. Conversely, Shastry et al. (2016) reported higher retentive force for ball and bar attachments over LOCATOR attachments; however, this discrepancy could be attributed to differences in study design, including the use of universal testing machines in their in vitro model, compared to our in vivo setup and the use of digital force gauge devices [2].

The present study also revealed that the self-aligning positioner attachments yielded higher mean bite force values at all measured time points. This may be due to their low-profile, dual-retention design and reduced resilience compared to the ball attachments, which allow greater prosthetic movement. Our findings are consistent with those of Helmy and Kothayer (2019), who also reported higher bite force with LOCATOR attachments [11]. In contrast, Bilhan et al. (2011) found no significant difference in bite force between various attachment types, likely due to differences in measurement techniques such as the use of strain gauges, whereas the present study employed a gnathodynamometer [12]. Patient satisfaction was significantly higher in Group II at one week, one month, and three months following prosthetic loading. The results of this study support the results obtained by Brandt et al. (2021), who found that patients with LOCATOR attachments had higher oral health-related quality of life (OHRQoL) than those with ball attachments [4]. However, studies by Krennmair et al. [13] and Chaware and Thakkar [5] observed no significant difference or varied preferences among patients depending on the attachment type, suggesting that individual patient expectations and clinical factors play a significant role. While the present study supports the clinical advantages of self-aligning positioner attachments over ball attachments, limitations such as small sample size and short follow-up period must be acknowledged. Additionally, factors such as parafunctional habits, implant inclination, inter-implant distance, and implant depth may influence outcomes. Therefore, further clinical research with larger sample sizes, extended follow-up periods, and comparisons across a wider variety of attachment systems are warranted to validate and expand upon these findings.

## Conclusions

The self-aligning positioner attachments are relatively better than ball attachments in terms of retention, bite force, and patient satisfaction. In conclusion, while the present study provides valuable insights into the comparison of attachment systems for implant-supported overdentures, further research is imperative to validate these findings comprehensively. Additional clinical studies focusing on other attachment systems are crucial for advancing the field of implant dentistry and to provide a better understanding of long-term outcomes of these attachments.
